# Search Behavior Regarding Cancer Susceptibility Genes Using a Clinical Decision Support Tool for Gene-Specific Penetrance: Content Analysis

**DOI:** 10.2196/28527

**Published:** 2021-07-13

**Authors:** Kanhua Yin, Jingan Zhou, Preeti Singh, Jin Wang, Danielle Braun, Kevin S Hughes

**Affiliations:** 1 Division of Surgical Oncology Massachusetts General Hospital Boston, MA United States; 2 Department of Surgery Harvard Medical School Boston, MA United States; 3 Department of General Surgery Beijing Anzhen Hospital Capital Medical University Beijing China; 4 Department of Breast Oncology Sun Yat-sen University Cancer Center State Key Laboratory of Oncology in South China, Collaborative Innovation Center of Cancer Medicine Guangzhou China; 5 Department of Data Science Dana-Farber Cancer Institute Boston, MA United States; 6 Department of Biostatistics Harvard T.H. Chan School of Public Health Boston, MA United States

**Keywords:** genetic testing, pathogenic variant, germline, risk communication, online health, digital health, cancer data, genetics, online tool, bioinformatics, web tool, cancer

## Abstract

**Background:**

Genetic testing for germline cancer susceptibility genes is widely available. The Ask2Me.org (All Syndromes Known to Man Evaluator) tool is a clinical decision support tool that provides evidence-based risk predictions for individuals with pathogenic variants in cancer susceptibility genes.

**Objective:**

The aim of this study was to understand the search behavior of the Ask2Me.org tool users, identify the patterns of queries entered, and discuss how to further improve the tool.

**Methods:**

We analyzed the Ask2Me.org user-generated queries collected between December 12, 2018, and October 8, 2019. The gene frequencies of the user-generated queries were compared with previously published panel testing data to assess the correspondence between usage and prevalence of pathogenic variants. The frequencies of prior cancer in the user-generated queries were compared with the most recent US population–based cancer incidence.

**Results:**

A total of 10,085 search queries were evaluated. The average age submitted in the queries was 48.8 (SD 16.5) years, and 84.1% (8478/10,085) of the submitted queries were for females. *BRCA2* (1671/10,085, 16.6%), *BRCA1* (1627/10,085, 16.1%), *CHEK2* (994/10,085, 9.9%), *ATM* (662/10,085, 6.6%), and *APC* (492/10,085, 4.9%) were the top 5 genes searched by users. There was a strong linear correlation between genes queried by users and the frequency of pathogenic variants reported in published panel testing data (*r*=0.95, r^2^=0.90, *P*<.001). Over half of the queries (5343/10,085, 53.0%) included a prior personal history of cancer. The frequencies of prior cancers in the queries on females were strongly correlated with US cancer incidences (*r*=0.97, r^2^=0.95, *P*<.001), while the same correlation was weaker among the queries on males (*r*=0.69, r^2^=0.47, *P*=.02).

**Conclusions:**

The patients entered in the Ask2Me.org tool are a representative cohort of patients with pathogenic variants in cancer susceptibility genes in the United States. While a majority of the queries were on breast cancer susceptibility genes, users also queried susceptibility genes with lower prevalence, which may represent a transformation from single gene testing to multigene panel testing. Owing to these changing tides, more efforts are needed to improve evidence-based clinical decision support tools to better aid clinicians and their practice.

## Introduction

Since the commercialization of *BRCA1* and *BRCA2* testing in 1996, the costs of DNA sequencing and genetic testing have dropped rapidly [1]. Today, germline multigene panel testing is widely used to assist cancer prevention and management. Based on genetic testing results, management strategies such as screening, surveillance, and risk-reducing surgery are now common in clinical guidelines and widely accepted by health care providers [2]. A recent study has shown that in managing patients with identified pathogenic variants, approximately 80% of the providers recommended clinical management aligned with the guidelines, and nearly all patients adhered to their providers’ recommendations [3]. Unfortunately, only 10%-15% of patients with breast and ovarian cancer in the United States who are eligible for genetic testing are actually tested [4], suggesting that there are still considerable gaps in the implementation of genetic testing.

One major challenge for clinicians in dealing with positive genetic testing results is to provide patients with accurate cancer risk estimates. Following genetic testing, patients usually rely on their providers to interpret results and assess cancer risk. However, literature regarding the magnitude of cancer risk for specific pathogenic variants (ie, penetrance) often varies in quality and study design. It is almost impossible for busy providers to keep up with the rapidly growing literature, carefully evaluate each study, and select the most reliable risk estimate [5,6]. In addition, despite the rapidly growing need, the availability of genetic counseling is still limited. In the United States, the estimated number of genetic counselors in 2018 was 4400, of whom only 48% practiced cancer genetics [7]. Patients, especially in rural areas, often have to wait weeks or months before seeing a genetic counselor. Therefore, an evidence-based, easily accessible, and regularly updated cancer risk prediction tool is needed to address these challenges.

The Ask2Me.org (All Syndromes Known to Man Evaluator) tool is a clinical decision support tool used for providing a summary of the major cancer susceptibility genes and the associated absolute cancer risk predictions [5,8]. Braun et al [5] describe the overall design and statistical basis of this tool, and it has been recommended as a resource in the American Society of Breast Surgeons hereditary breast cancer guidelines [9]. In this study, we aimed to understand the search behavior of Ask2Me.org users, identify the patterns of queries entered, and discuss how to further improve the tool.

## Methods

### User-Generated Queries

The Ask2Me.org tool allows users to enter patient information that includes their age and sex, prior surgical (bilateral mastectomy, hysterectomy, and oophorectomy) and cancer history, and select a gene with a pathogenic variant. In return, this tool provides a summary of that gene along with the patient’s future risk for each type of cancer associated with a pathogenic variant in the selected gene. A total of 35 genes can be queried in the Ask2Me.org tool, which covers most of the commonly tested cancer susceptibility genes such as *APC, ATM, BRCA1/2, CDH1, CHEK2, PTEN, STK11,* and *TP53*. Personal health information such as name, date of birth, home address, and email address is not collected when using this tool. For this study, we collected user-generated queries from the Ask2Me.org tool between December 12, 2018, and October 8, 2019. Queries correspond to test cases, research use, and real patients. From each query, we collected the age, sex, genes with the pathogenic variant, prior cancer, and surgical history (bilateral mastectomy, hysterectomy, and oophorectomy).

### Reference Groups

To assess the correlation between the frequency of genes entered by users in the Ask2Me.org tool and the frequency of pathogenic variants among patients who undergo panel testing (ie, the targeted user group of this tool), we used a large multigene cancer panel cohort reported by LaDuca et al [10] as the reference group. Based on 165,000 patients undergoing hereditary cancer predisposition testing between 2012 and 2016 at a single diagnostic laboratory, LaDuca et al’s study reported the frequency of pathogenic variants across 32 cancer susceptibility genes. Their cohort’s median age was 52 (IQR 43-62) years, which was similar to that in this study (median 49 [IQR 37-61] years). The majority of the included patients were females (94.2%), Caucasian (64.0%), and had a personal history of cancer (72.5%) or a history of family history of cancer among first-degree and second-degree relatives (90.1%) [10]. To access the correlation between the frequency of prior cancer in the Ask2Me.org user–generated queries and the cancer incidence at the population level, we used the most recent US population–based cancer incidence estimated by the American Cancer Society [11].

### Statistical Analysis

Continuous data were expressed as mean (SD) and median (IQR). Categorical data were expressed as percentages. Pearson correlation coefficients were used to evaluate the degree of correlation between both user-generated queries and published gene frequencies and user-generated queries and population-based cancer incidence. A linear regression model was fitted to visualize the results. As the default setting of the Ask2Me.org tool—a 25-year-old female with no cancer or surgical history as a likely test case—a sensitivity analysis was performed by excluding these entries and re-evaluating the correlations. *P* values less than .05 were considered statistically significant. All analyses were performed using the R language statistical software (version 4.0.3; R Foundation for Statistical Computing).

## Results

### Queried Susceptibility Genes

**Figure 1:**
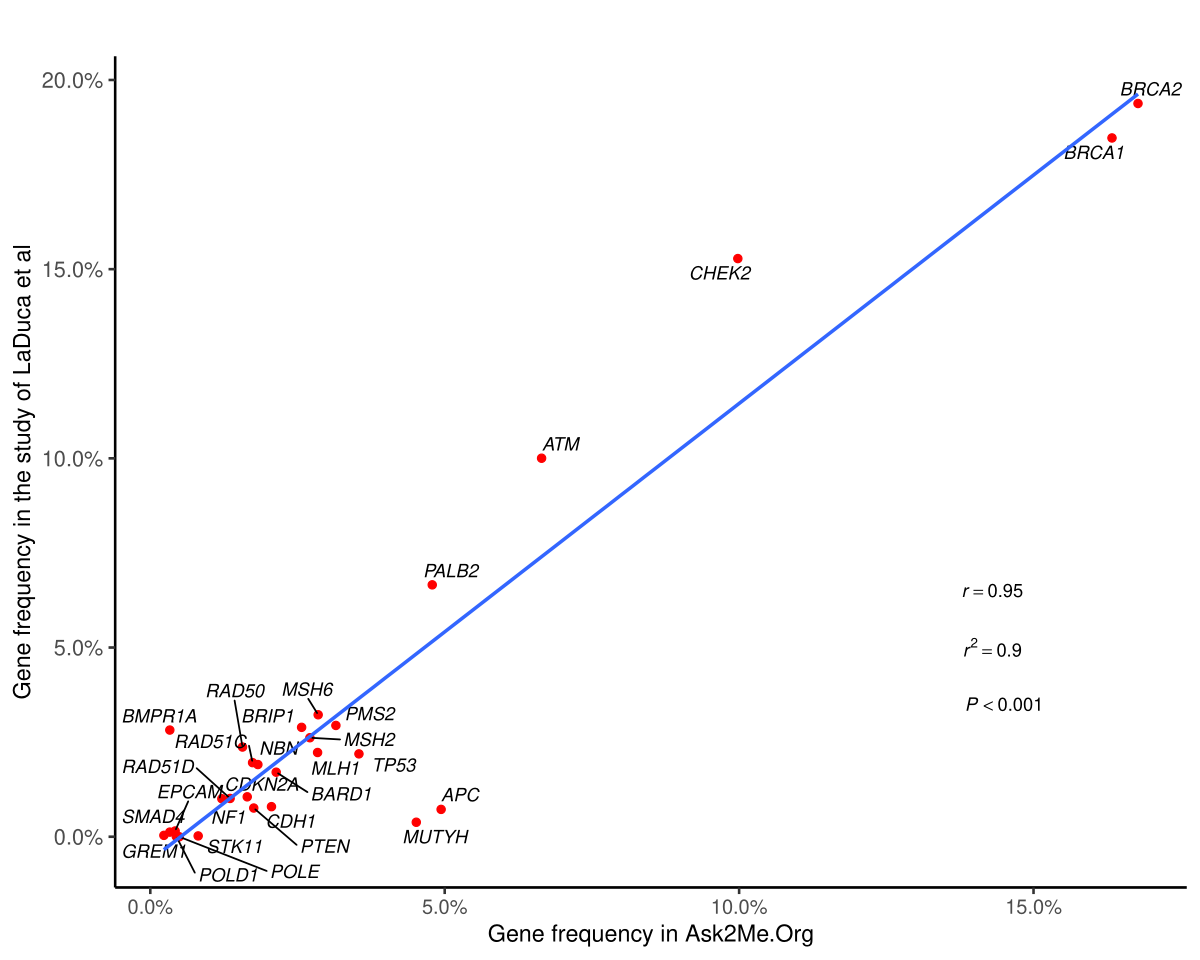
Correlation between frequencies of genes entered by the Ask2Me.org tool users and frequencies of pathogenic variants in panel testing results reported by LaDuca et al [10]. The blue line represents the results from the regression model.

From December 12, 2018 to October 8, 2019 (300 days), 10,085 queries were submitted to the Ask2Me.org tool. The average age submitted in the query was 48.8 (SD 16.5) years (median 49 [IQR 37-61] years), and 84.1% (8478/10,085) of the submitted queries were for females. *BRCA2* (1671/10,085, 16.6%), *BRCA1* (1627/10,085, 16.1%), *CHEK2* (994/10,085, 9.9%), *ATM* (662/10,085, 6.6%), and *APC* (492/10,085, 4.9%) were the 5 most common genes searched by users. Lynch syndrome–associated genes such as *PMS2* (314/10,085, 3.1%), *MSH6* (284/10,085, 2.8%), *MLH1* (283/10,085, 2.8%), *MSH2* (270/10,085, 2.7%), and *EPCAM* (42/10,085, 0.04%) were queried less frequently. Seven out of the ten most commonly searched genes were breast cancer susceptibility genes. For queries on females (8478/10,085, 84.1%; mean age 49.2 [SD 16.1] years; median age 50 [IQR 38-61] years), 79.7% (6757/8478) of the queries were of breast cancer susceptibility genes, and the top 5 genes were *BRCA1* (1467/8478, 17.3%), *BRCA2* (1449/8478, 17.1%), *CHEK2* (895/8478, 10.6%), *ATM* (548/8478, 6.5%), and *PALB2* (402/8478, 4.7%). For queries on males (1607/10,085, 15.9%; mean age 46.4 [SD 18.4] years; median age 47 [IQR 32-61] years), the top 5 genes queried were *BRCA2* (222/1607, 13.8%), *BRCA1* (160/1607, 10.0%), *ATM* (114/1607, 7.1%), *APC* (105/1607, 6.5%), and *CHEK2* (99/1607, 6.2%). Comparing the top 10 genes queried on females and males, we found 8 of them overlapped, namely, *APC, ATM, BRCA1, BRCA2, CHEK2, MUTYH, PALB2,* and *TP53. BRIP2* and *PMS2* were only listed in the top 10 queries on females, and *MLH1* and *MSH2* were only listed in the top 10 queries on males. After excluding 3 individual queries without age information, we found that there were 2979 (29.5%), 4422 (43.9%), and 2681 (26.6%) queries in the <40 years, 40-60 years, and >60 years age groups, respectively. In the <40 years age groups, the top 5 queried genes were *BRCA1* (479/2979, 16.1%), *BRCA2* (383/2979, 12.9%), *CHEK2* (215/2979, 7.2%), *APC* (215/2979, 7.2%), and *ATM* (173/2979, 5.8%). In the 40-60 years age group, the top 5 queried genes were *BRCA2* (757/4422, 17.1%), *BRCA1* (683/4422, 15.4%), *CHEK2* (469/4422, 10.6%), *ATM* (265/4422, 6.0%), and *PALB2* (208/4422, 4.7%). Similarly, in the >60 years age group, the top 5 queried genes were still *BRCA2* (530/2681, 19.8%), *BRCA1* (464/2681, 17.3%), *CHEK2* (310/2681, 11.6%), *ATM* (224/2681, 8.4%), and *PALB2* (141/2681, 5.3%). There was a strong linear correlation between the frequencies of genes entered by users in the Ask2Me.org tool and the frequencies of pathogenic variants reported by LaDuca et al (*r*=0.95, r^2^=0.90, *P*<.001; Figure 1) [10]. By excluding the queries with the default setting (ie, 25-year-old female, no prior cancer, and no history of surgery), the strong linear correlation was still maintained (*r*=0.95, r^2^=0.91, *P<*.001).

### Prior History of Cancers

**Figure 2:**
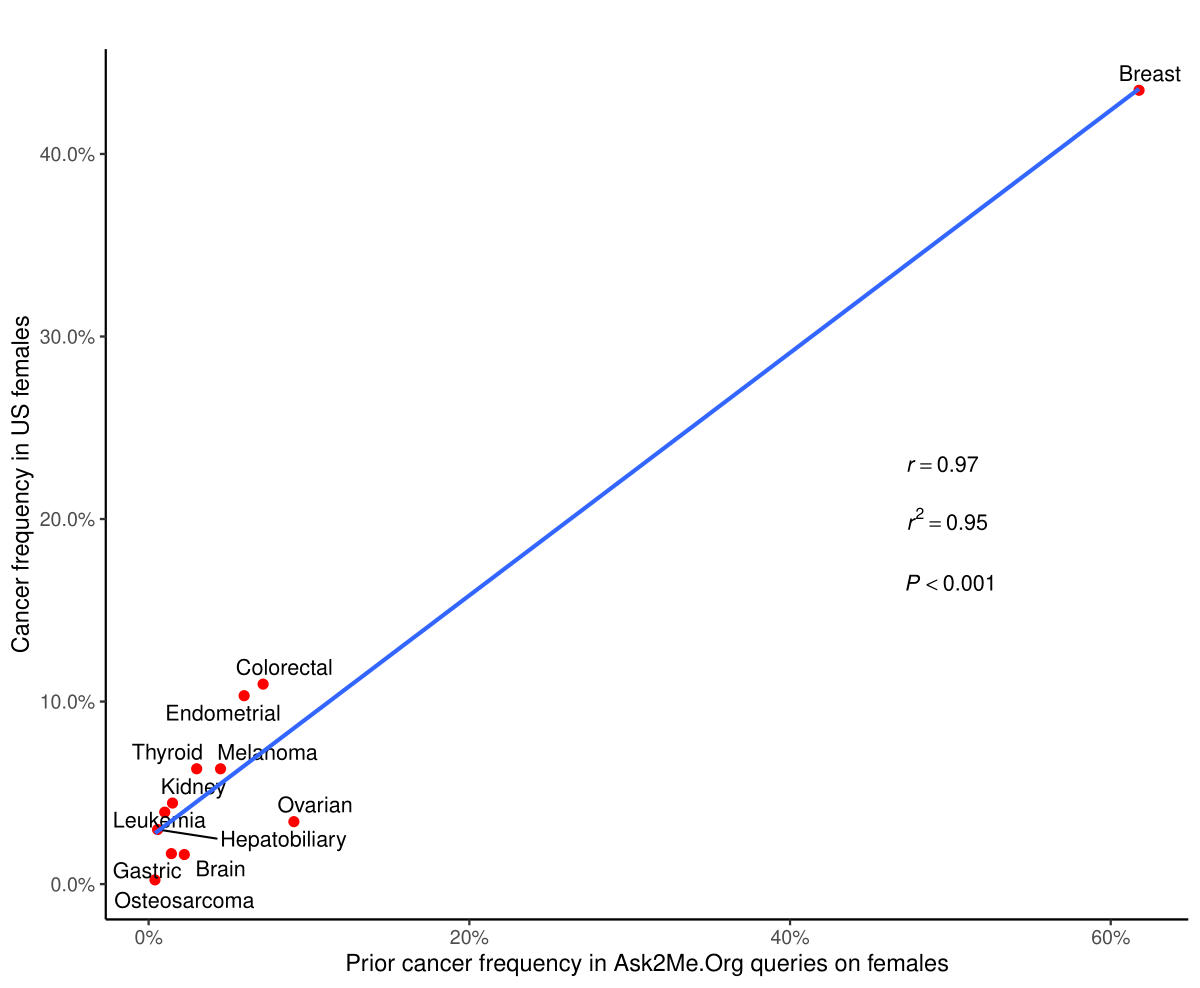
Correlation between frequencies of prior cancers in the Ask2Me.org user queries on females and corresponding US cancer incidences. The blue line represents the results from the regression model.

**Figure 3:**
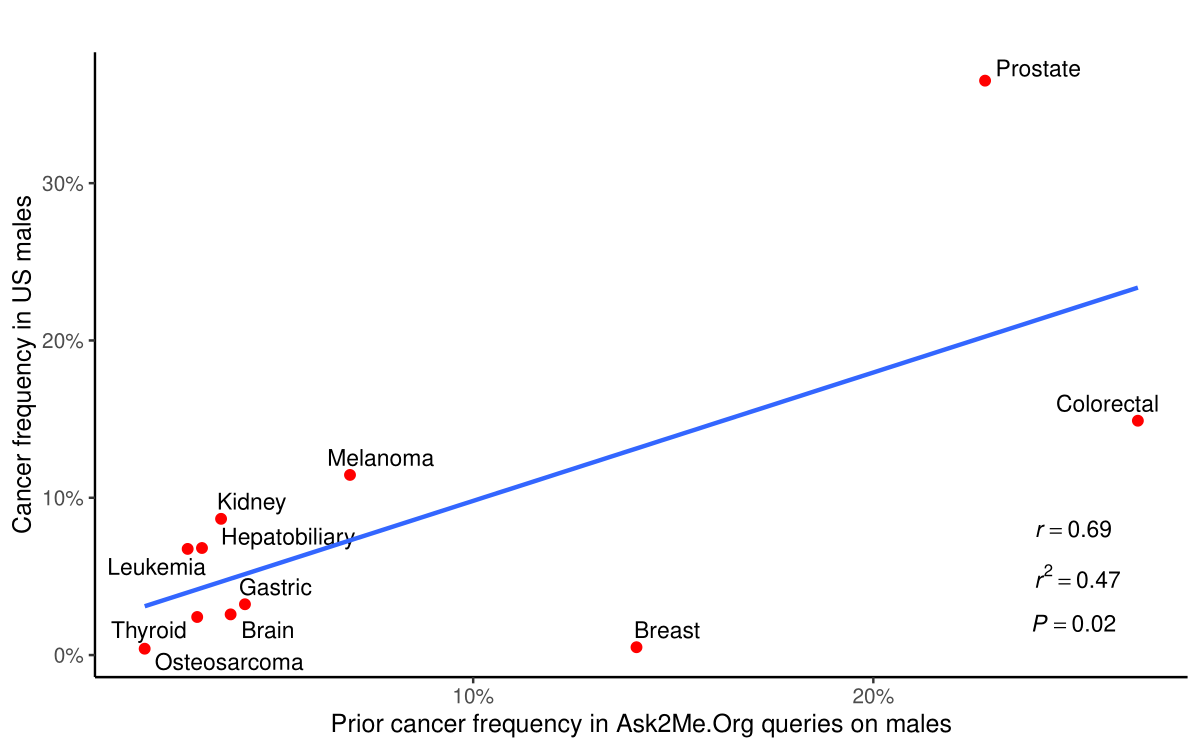
Correlation between frequencies of prior cancers in the Ask2Me.org user queries on males and corresponding US cancer incidences. The blue line represents the results from the regression model.

Of the 10,085 queries, 5343 queries (52.9%) entered a prior history of cancer, comprising 56.3% (4771/8478) of the queries on females and 35.6% (572/1607) of the queries on males. The frequencies of the type of prior cancer in the queries on females have a strong linear correlation with the corresponding US cancer incidences (*r*=0.97, r^2^=0.95, *P<*.001; Figure 2), while the same correlation was weaker in the queries on males (*r*=0.69, r^2^=0.47, *P*=.02, Figure 3). Sensitivity analysis revealed that the above linear correlation in queries on females did not change significantly after excluding the queries with the default setting (females: *r*=0.97, r^2^=0.95, *P<*.001; the correlation in males was not affected by removing the queries with the default setting). There were 634 queries (11.9% of all queries with cancer history) who selected multiple prior cancers. Among the 521 queries on females with multiple cancers, breast and ovarian cancers (78/521, 15.0%) and breast cancer and melanoma (70/521, 13.4%) were the 2 most common combinations. Among the 113 queries on males with multiple cancers, prostate and colorectal cancers were the most common combination (42/113, 37.2%). In addition, 23.0% (1947/8478) of queries were on females who had an oophorectomy, 22.4% (1899/8478) of queries were on females who had a hysterectomy, and 17.2% (1462/8478) of queries were on females who had a bilateral mastectomy.

## Discussion

In this study, by analyzing over 10,000 user queries, we characterized the search behaviors of Ask2Me.org tool users and identified the patterns of pathogenic variants and cancer history among the queries. We found that breast cancer susceptibility genes were the most commonly searched genes in both males and females. There was a strong linear correlation between the frequencies of genes entered by Ask2Me.org users and the prevalence of pathogenic variants in panel testing results recently reported by LaDuca et al [10]. Over half of the queries included a prior cancer history. The frequencies of prior cancers in the queries on females had a strong correlation with US cancer incidences, while the same correlation was weaker among the queries on males. Overall, these findings suggest that the patients entered into the Ask2Me.org tool are a representative cohort of patients with pathogenic variants in the United States. We found that the majority of the commonly searched genes are breast cancer susceptibility genes. In current practice, most germline genetic test takers are individuals who have a high suspicion of hereditary cancer predisposition. Among all types of cancers, breast cancer’s inherited component is one of the most intensively studied and appreciated. Several breast cancer risk assessment models have been developed to identify individuals with a high risk of being pathogenic variant carriers [12] and they are widely implemented in clinical practice. Taking all this together, plus the high incidence of breast cancer (30% of all newly diagnosed cancers in US women) [11], it is not surprising to see the majority of the commonly searched genes are related to breast cancer. In contrast, we found that genes associated with colorectal cancer were searched less frequently, with none of the 6 Lynch syndrome–associated genes accounting for more than 3.9% (400/10,085) of the total searches. This may be due to the less frequent use of multigene panel testing in patients with colorectal cancer and their families. With the inclusion of more newly identified genes in the National Comprehensive Cancer Network colorectal cancer genetic/familial high-risk assessment guidelines [13] and more wide use of panel testing in clinical practice, we expect to see an increasing search of colorectal cancer susceptibility genes in the Ask2Me.org tool in the future.

Hart et al [14] recently reported the pathogenic variant prevalence among nearly 148,000 individuals referred for hereditary cancer genetic testing. The most prevalent mutated genes in this high-risk population share a similar pattern as we identified in the Ask2Me.org tool: 8 out of the top 10 most frequently mutated genes found in these 148,000 individuals are among the top 10 most commonly searched genes in the Ask2Me.org tool. In addition, there was a strong linear relationship between the frequencies of genes entered by users in the Ask2Me.org tool and the frequencies of pathogenic variants in the panel testing results reported by LaDuca et al [10]. These findings suggest that the rates of queries in the Ask2Me.org tool may be proportional to their prevalence. Users not only queried commonly tested genes such as *BRCA1* and *BRCA2* but also queried lower prevalence genes, which may represent the shift from single gene testing to multigene panel testing.

Over half of queries entered in the Ask2Me.org tool included a personal history of cancer, with around 10% of them having multiple cancers. These results show that queries with prior cancers accounted for a considerable portion of the Ask2Me.org tool user queries. Similarly, in LaDuca et al’s 165,000-patient cohort, 72.5% of patients had a personal history of cancer [10]. This is likely in part because most patients tested already have cancer [15]. As Dr. Mary-Claire King stated at her Lasker Award speech, this represents “a failure of cancer prevention” [16]. These findings suggest that we need to increase genetic testing in people who do not yet have cancer and implement appropriate interventions before cancer develops. We also observed a strong linear association between the frequencies of queries with prior cancers entered and the US cancer incidences in queries on females, further demonstrating that queries on females were not only limited to one or several cancer types but distributed proportionally to the population-level cancer incidence. The same correlation in queries on males was weaker, which may be explained by the relatively young age entered in these male queries (median age 47 years). Since approximately 60% of prostate cancer cases are diagnosed in men older than 65 years [17], the young age in Ask2Me.org male queries may result in a lower proportion of prostate cancers queried compared to the US population–based incidence of prostate cancers.

The indications for germline genetic testing have been expanded in recent decades. In addition to testing for hereditary breast and ovarian cancer, germline genetic testing has also been recommended to manage other cancers such as colorectal cancer, pancreatic cancer, and prostate cancer. It is essential to incorporate high-quality, evidence-based, and easy-to-access clinical decision support tools into the interpretation of testing results and the personalization of disease prevention and clinical management plans. One purpose of studying search behavior is to understand user needs and further improve the Ask2Me.org tool. Since this tool became available in 2016, efforts have been made to optimize and improve this clinical decision support tool. A natural language processing algorithm was developed to classify medical literature on cancer susceptibility genes [18]. Based on this algorithm, a semiautomated natural language processing–based procedure was developed to identify the penetrance studies in the medical literature, which has proven to reduce 84% of the abstract review workload and cover 99% of penetrance studies [6,19]. In addition, we have reviewed over 10,000 cancer genetic papers identified over 700 penetrance studies and provided the absolute risk curves for at least 154 gene-cancer combinations. In addition, a framework of the systematic review and verification of gene-disease associations has been developed [20]. Using this framework, we have examined all genes listed in the Ask2Me.org tool, verified over 500 gene-disease associations, and reported the disease spectra for breast, thyroid, and gastric cancer susceptibility genes [20-23]. Since users are not only interested in the commonly tested genes such as *BRCA1/2*, we plan to expand the Ask2Me.org tool to cover a broader range of genes, especially those with relatively low prevalence. Further, as over half of the queries included a prior personal history of cancer, we hope in the future that cascade testing will allow patients with no cancer to benefit from increased surveillance. Moreover, additional cancer-related features such as infection status (eg, HPV, HIV) and cancer status (eg, remission, recurrence) may also be incorporated into this tool.

This study has several limitations. First, search queries are likely to correspond to not only real patients but also test cases or research purposes. Although we performed sensitivity analyses by removing the queries with default settings, there is still no way to explicitly distinguish them. Second, the frequencies of genes and prior cancers were only reflective of the search queries of the Ask2Me.org tool but may not represent the actual prevalence of the pathogenic variants and cancers at the population level. Third, as the vast majority of users are in the United States, the current findings in the searching behavior may not be generalized to users from other countries. The Ask2Me.org tool has become an increasingly recognized clinical decision tool that provides risk predictions for patients with pathogenic variants in cancer susceptibility genes. There is a strong linear relationship between the frequencies of genes entered by the Ask2Me.org tool users and the frequencies of pathogenic variants in panel testing results reported by LaDuca et al [10]. The frequencies of prior cancers in the queries on females have a strong correlation with US cancer incidences, while the same correlation was weaker among the queries on males. Our data suggest that clinicians seek information on almost all genes identified and not just the less recognized or more recently identified genes.

## Abbreviations

Ask2Me: All Syndromes Known to Man Evaluator
